# Training Emergency Physicians in Ultrasound-guided Fascia Iliaca Compartment Blocks: Lessons in Change Management

**DOI:** 10.7759/cureus.4773

**Published:** 2019-05-28

**Authors:** Juliana Wilson, Kaylah Maloney, Kelly Bookman, Jason W Stoneback, Vaughn A Browne, Adit Ginde, Mary Wallace, Gabrielle Jacknin, Ethan Cumbler, Resa E Lewiss

**Affiliations:** 1 Emergency Medicine, University of Colorado, Denver, USA; 2 Emergency Medicine, Thomas Jefferson University, Philadelphia, USA; 3 Emergency Medicine, University of Colorado, Aurora, USA; 4 Orthopedics, University of Colorado, Aurora, USA; 5 Internal Medine, University of Colorado, Aurora, USA; 6 Pharmacy, University of Colorado Hospital, Denver, USA; 7 Internal Medicine, University of Colorado, Aurora, USA

**Keywords:** regional anesthesia, point of care ultrasound, change management, emergency medicine

## Abstract

Study objectives

Older adults who sustain hip fractures are susceptible to high rates of morbidity and mortality. The systemic administration of opioids is associated with side effects disproportionately affecting the elderly. The ultrasound-guided fascia iliaca compartment block procedure (FICB) is associated with a reduced patient need for oral and parenteral opioids and with improved functional outcomes. We designed a multi-disciplinary quality improvement initiative to train emergency physicians (EPs) to perform the ultrasound-guided FICB procedure for geriatric hip fracture patients. We examined the lessons derived from the EPs' resistance to implementing a practice-changing behavior.

Methods

This study was a prospective observational cohort study. We included all emergency department (ED) patients > 65 years with X-ray confirmation of isolated hip fractures. We also enrolled the treating EPs. Patients were enrolled from March 2016 to January 2017 in an urban, academic ED with 100,000 annual visits. The ED ultrasound faculty trained ED faculty and residents in the FICB procedure. Seventeen of 50 attending EPs completed the training: classroom lecture and online narrated video instruction. The hands-on sessions consisted of three stations: scan a human model volunteer to review the sonoanatomy, practice the needle technique using a Blue Phantom^TM^ Regional Anesthesia Ultrasound Training Block Model (Simulaids, Inc., NY, US), and practice the needle technique using a static simulator. We created a multi-disciplinary geriatric hip fracture order set for the electronic medical record. The attending EPs, caring for eligible patients, were asked to complete a Research Electronic Data Capture (REDCap) survey, and we analyzed the data using descriptive statistics.

Results

We enrolled 77 geriatric hip fracture patients. Two of the 77 patients received FICB. Thirty-two EPs participated as providers for these patients while 97% of these providers completed the post-intervention survey. Providers used the geriatric hip fracture order set in 10 of 77 encounters. Most EPs did not perform the block because they were not trained or did not feel comfortable performing it.

Conclusion

Despite the efficacy supported by the literature and training sessions offered, the EPs in this study did not adopt the FICB procedure. Future efforts could include developing a FICB on-call team, increasing the proportion of trained EPs through initial supervised hands-on practice, and partnering financial or education incentives with getting trained.

## Introduction

Older adults who sustain hip fractures are susceptible to high rates of morbidity and mortality [1]. Pain control for this population is difficult because opioids, the most commonly used medications, are associated with safety concerns, such as respiratory depression, altered mental status, and an increased incidence of falls. Regional anesthesia performed under direct ultrasound visualization provides excellent anesthesia, a reduced need for oral and parenteral opioids, and improved functional outcomes [2-3]. We designed a multi-disciplinary quality improvement initiative to train emergency physicians (EPs) in ultrasound-guided fascia iliaca compartment blocks (FICB). The original intent of the study was to compare demographics, length of stay, total opiate and non-opiate pain medications used, as well as outcomes between geriatric patients that received the FICB and those that did not. As we implemented the training and study and began data collection, we found that few FICBs were being performed. In response to this finding, the project pivoted to understanding barriers to the adoption of this new skill. We examined the lessons in change management derived from the EPs resistance as well as barriers to implementing a quality improvement program and changing the EPs' behavior.

## Materials and methods

We studied all emergency department (ED) patients greater than 65 years of age, with radiographic confirmation of isolated hip fracture and who were able to give written informed consent. Patients were enrolled from March 2016 to January 2017 in an urban, academic ED with 100,000 annual visits. We also enrolled the treating EPs. The ED ultrasound faculty organized voluntary training sessions following faculty department meetings that consisted of human model scanning, Blue Phantom^TM^ Regional Anesthesia Ultrasound Training Block station (Simulaids, Inc., NY, US), and a muscle nerve fascia static simulator [4]. There were flipped classroom online reading assignments, an online narrated training video, and a portable document format (.pdf) document available to all EPs. The images used in the training sessions are given in the Appendix.

Seventeen of 50 attending EPs completed the training. A geriatric hip fracture order set was created in the electronic medical record. This standardized the equipment, medications, and tests ordered for the procedure. This also minimized EPs missing certain orders prior to the nerve block. To simplify the process, we pre-filled individual plastic bags, containing all items needed to perform the procedure. For each geriatric hip fracture patient, we collected demographic information, hip fracture characteristics, pain medication administered, and hospital length of stay. We collected prospective data and performed a descriptive analysis of the ED attending physicians, the procedure performance, and the order set utilization. The attending EPs, caring for eligible patients, were asked to complete a Research Electronic Data Capture (REDCap) survey. This REDCap survey assessed which EPs performed the block, those who did not, the reasoning for not performing, and whether or not they used the order set. We included the use of the order set as it was designed to decrease barriers to the implementation of the FICB by bundling orders for medications, neurologic checks, and monitoring. For this study, R, version 3.4.0, was used for all statistical analysis. The mean and standard deviation were reported for continuous data and percentages were reported for categorical data.

## Results

There were 77 geriatric patients with hip fractures that met inclusion criteria for enrollment (Table 1).

**Table 1:** Shows the demographic data for the 77 patients enrolled during the study period

Variable	All Patients (n = 77, 100%)	Block Patients (n = 2, 2.60%)	No Block Patients (n = 75, 97.40%)
Age (years) (mean ± sd)	81.32 ± 9.28	79.5 ± 10.61	81.37 ± 9.32
Older than 90 (%)	22.08	0	22.67
Sex (%)			
Male	29.87	50.00	29.33
Female	70.13	50.00	70.67
Race (%)			
Caucasian	79.22	100	78.67
African American	12.99	0	13.33
Asian	2.60	0	2.67
Other	5.19	0	5.33
Hispanic (%)			
Yes	9.09	0	9.33
No	90.91	100	90.67

**Figure 1:**
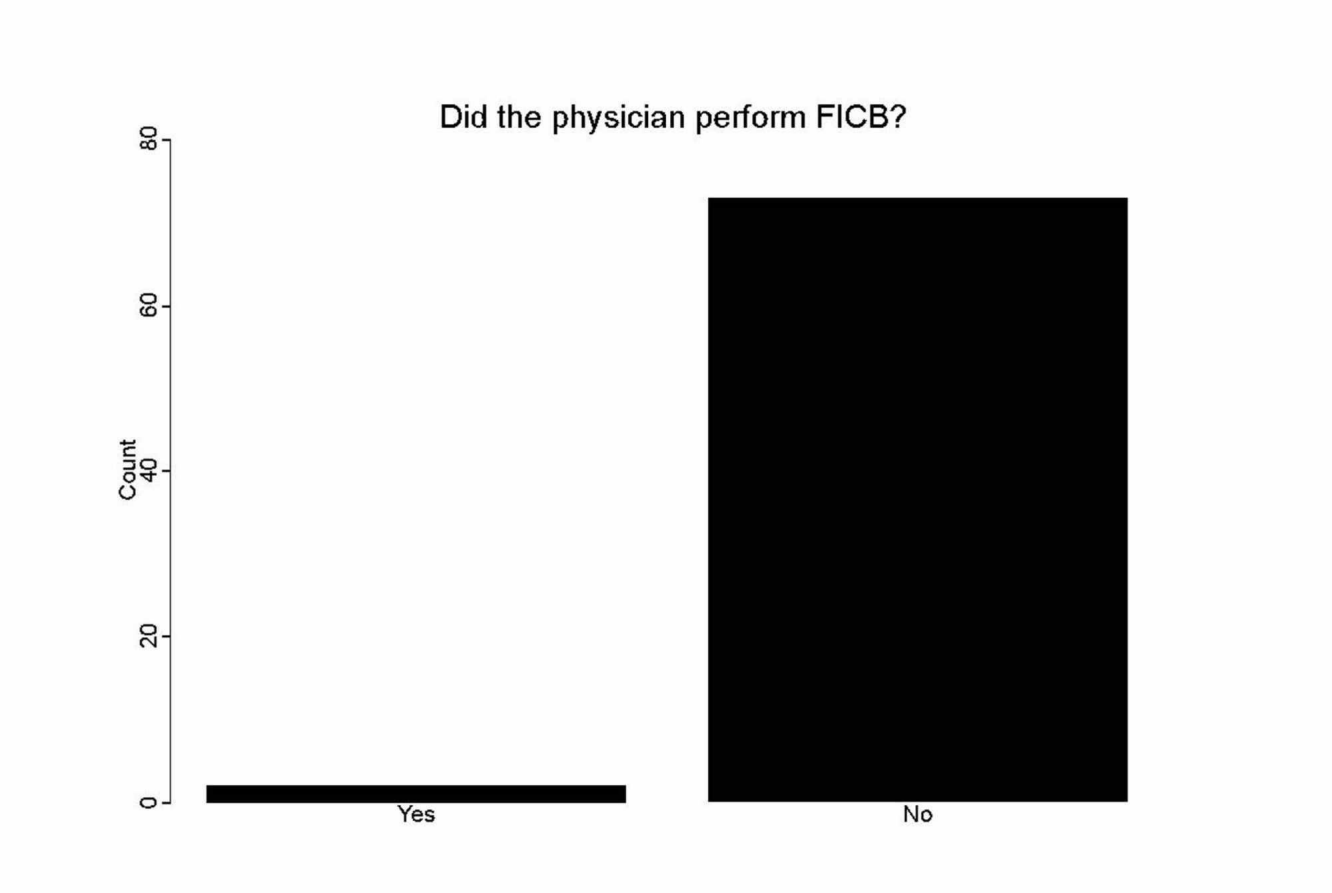
Ratio of physicians who performed the fascia iliaca compartment block (FICB)

Thirty-two treating EPs participated in the study. The EPs completed a survey for each geriatric hip fracture patient that they treated. We had a greater than 97% response rate to the post-intervention survey. Of the 77 patients, two received FICB (Figure 1).

**Figure 2:**
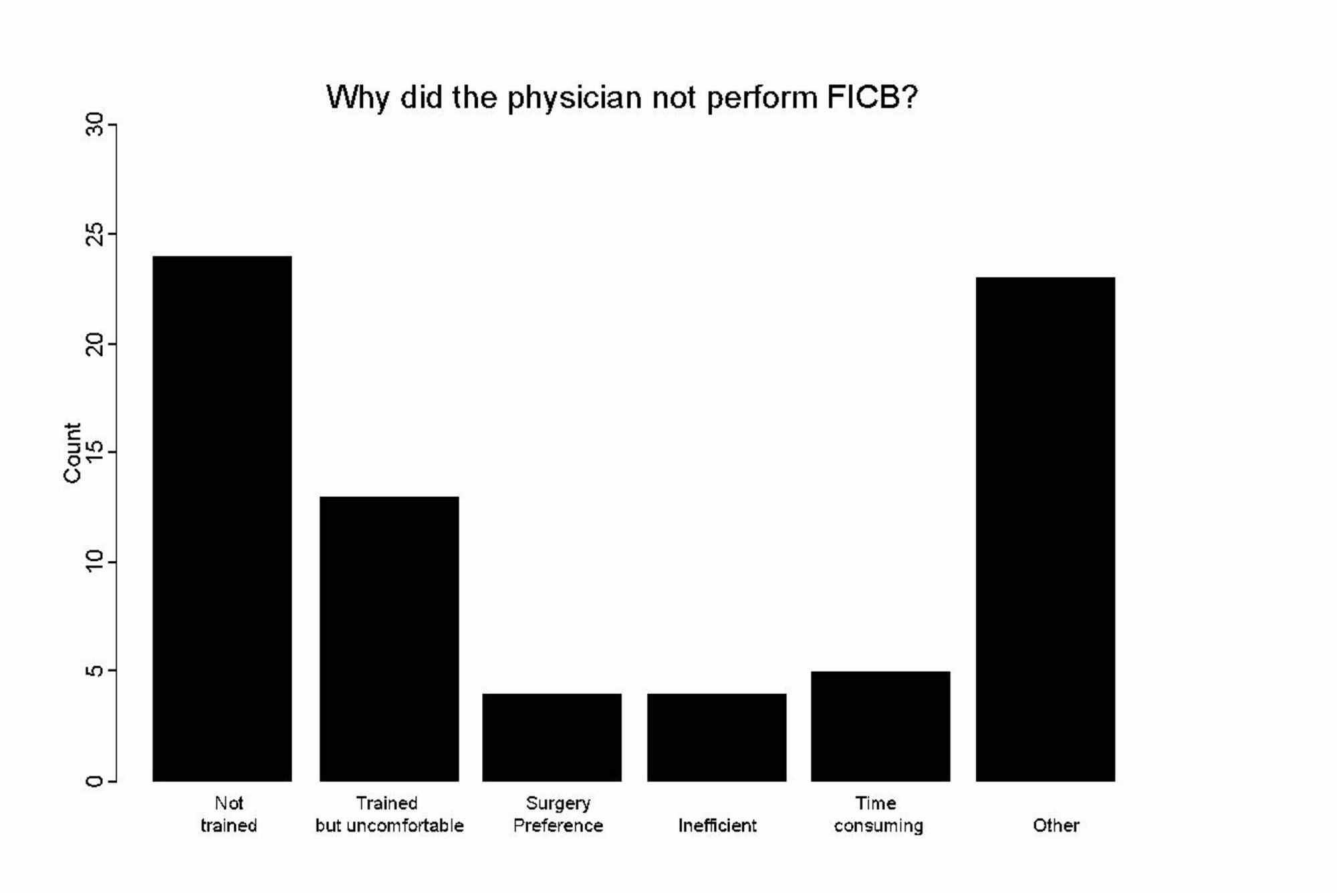
Reasons the EP did not perform the fascia iliaca compartment block (FICB)

The more common reasons that physicians did not perform FICB: not trained (24 of 77: 31%) and not comfortable performing FICB, despite completing the training (13 of 77: 17 %) (Figure 2).

**Figure 3:**
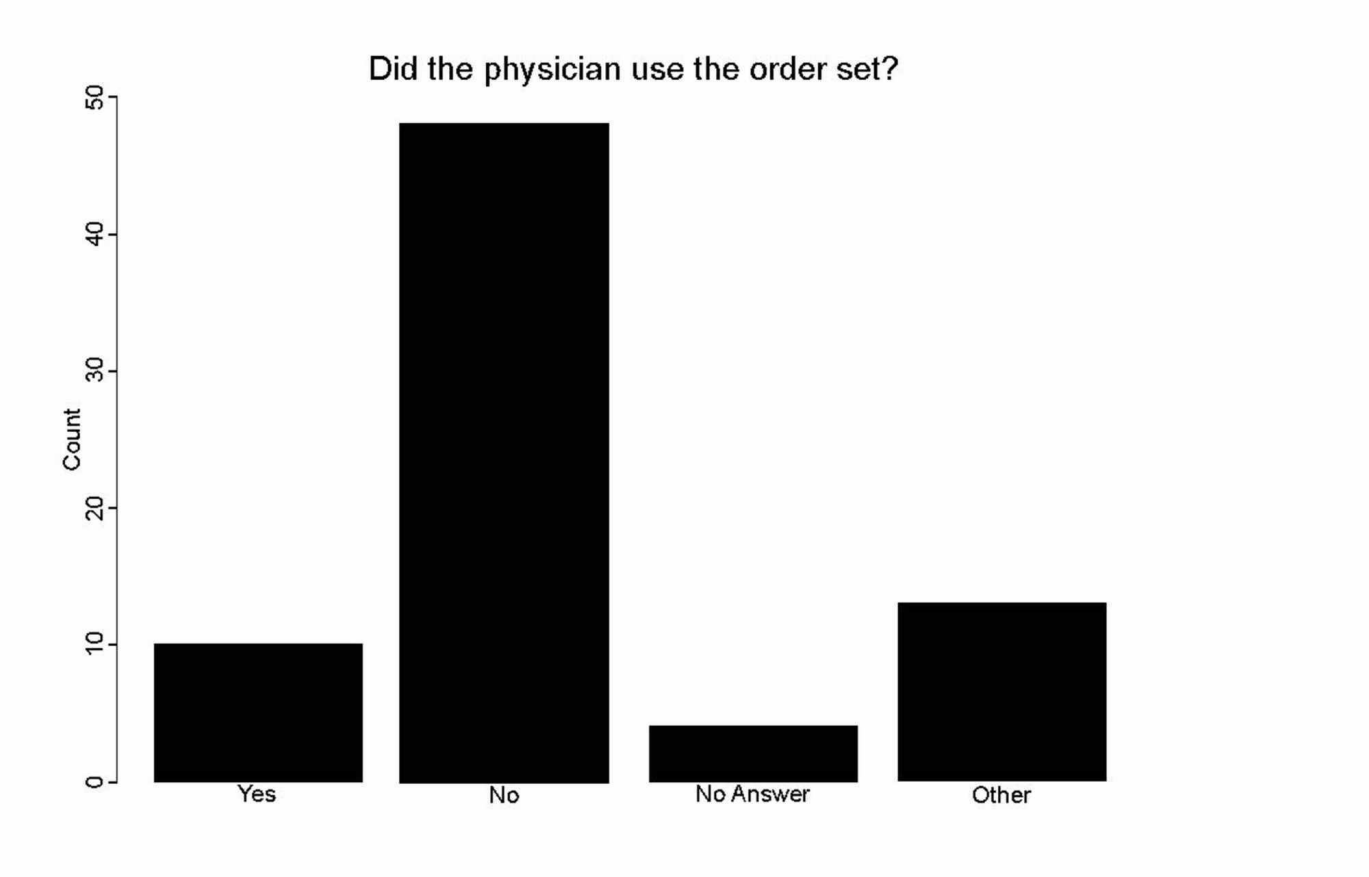
Ratio of physicians who used the geriatric hip fracture order set

A few of the physicians (10 of 77: 12.99% ) used the ED geriatric hip fracture order set (Figure 3).

The two patients who received FICB had a shorter total length of stay overall in the hospital as compared to the no block patients (82.38 and 143.54 hours, respectively) (Table 2).

**Table 2:** Demographic information, admitting service and disposition of geriatric hip fracture patients

Variable	All Patients (n = 77, 100% )	Block Patients (n = 2, 2.60%)	No Block Patients (n = 75, 97.40%)
Height ( meters) (mean ± sd)	1.66 ± 0.12	1.73 ± 0.07	1.65 ± 0.12
Weight (kg)(mean ± sd)	63.51 ± 15.21	63.25 ± 17.61	63.52 ± 15.28
Body Mass Index (mean ± sd)	23.12 ± 4.81	20.95 ± 4.16	23.18 ± 4.83
Length of Stay-ED (hours ) (mean ± sd)	5.37 ± 5.27	5.05 ± 0.04	5.38 ± 5.34
Pre-Operation Time (hours) (mean ± sd)	25.41 ± 14.4	24.82 ± 1.59	25.43 ± 14.62
Length of Stay-Total (hours) (mean ± sd)	141.93 ± 140.75	82.28 ± 18.6	143.54 ± 142.3
Admitting Service (%)			
Acute Care Elderly	1.30	0	1.33
Acute Care Surgery-Trauma	7.79	0	8.00
General Surgery	1.30	0	1.33
Hospitalist-Hospital Medicine Service	1.30	0	1.33
Medicine 2	1.30	0	1.33
Medicine 3	1.30	0	1.33
Orthopedic Surgery	54.55	100	53.33
Orthopedics	23.38	0	24.00
Vascular Surgery	1.30	0	1.33
Unknown	6.49	0	6.67
Hip Fracture Diagnosis (%)	96.10	100	96.00
Discharge Disposition (%)			
Discharge to another facility	1.30	0	1.33
Home/Hospice	24.68	0	25.33
Rehab Facility	6.49	50	5.33
Skilled Nursing Facility	67.53	50	68.00
Order Set Performed (%)	19.48	100	17.33
Fracture Type (%)			
Femoral neck	35.14	0	36.11
Intertrochanteric Fracture	50.00	100	48.61
Intracapsular Fracture	1.35	0	1.39
Subtrochanteric Fracture	4.05	0	4.17
Unknown	9.46	0	9.72

## Discussion

This quality improvement study can be viewed as a lesson in change management. Although there have been many studies conducted to explore aspects of practice change, implementing change in healthcare remains challenging [5-6]. Part of change management is forming a powerful guiding coalition and creating and communicating a vision [7-9]. Change management and the implementation of a new practice also involves several steps. These steps include: (1) knowledge, which includes the initial step of educating and communicating to staff; (2) persuasion or using incentives to encourage staff involvement; (3) decision or the staff making the choice to accept change; (4) implementation of the new practice; and (5) confirmation in which the staff acknowledges the benefits of the practice change [10]. There were several steps that presented as roadblocks in the implementation of the FICB in this study. The initial step in education was accomplished by the multimodal training implemented. However, some EPs stated that they were uncomfortable performing the FICB independently despite attending training. The second step of persuasion or incentivizing staff also posed an obstacle as translating the enthusiasm of the early adopting EPs to influence more of the majority was a critical challenge in this initiative. Without the mindset that the FICB would be a great change within the department, the adoption of this skill was an obstacle. In future implementations, there are at least two ways in which department leadership could encourage more EP involvement: incentivizing or mandating. With the obstacles presented in the first two steps of the implementation, we were unable to proceed to further steps. The limitations of this study include the single urban academic center setting. The clinical environment is very time-efficient so arguably more FICBs would be performed if patients were in the ED longer. A second limitation was our difficulty in motivating faculty to attend the training. Perhaps if mandated or incentivized, more EPs would have been trained.

## Conclusions

We sought to study the impact of implementing a FICB treatment program for geriatric hip fracture patients. We found that we did not anticipate the barriers to the change management of the EP practice. In the future, anticipating these challenges while implementing a quality improvement initiative should aid in its success.

## Figures and Tables

**Figure 4:**
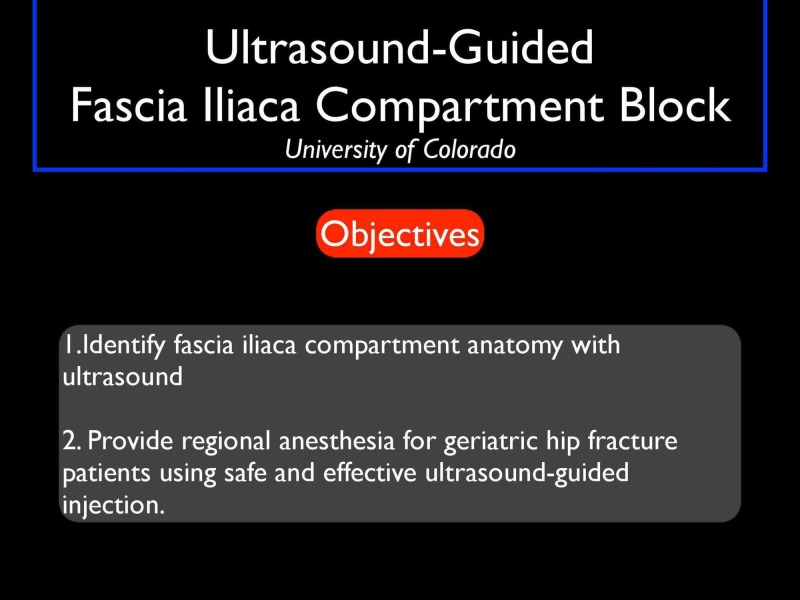
Image 1/12 of training presentation

**Figure 5:**
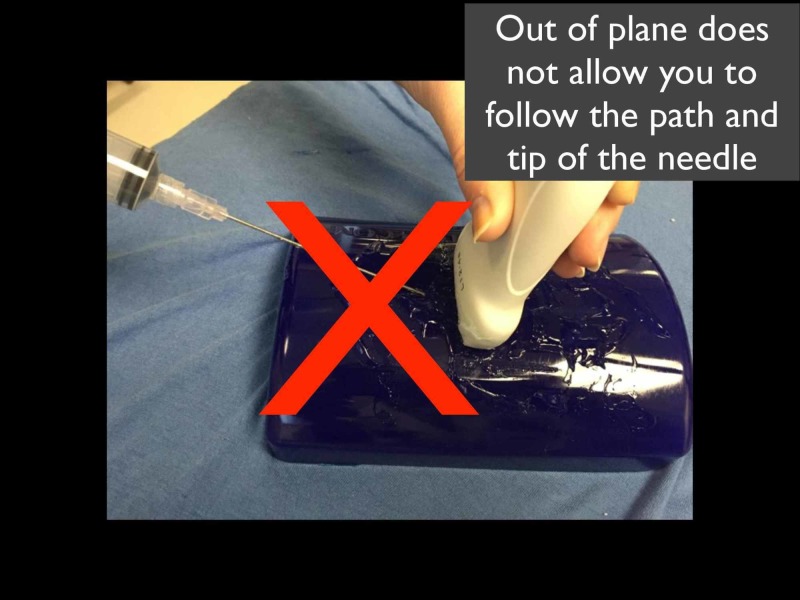
Image 2/12 of training presentation

**Figure 6:**
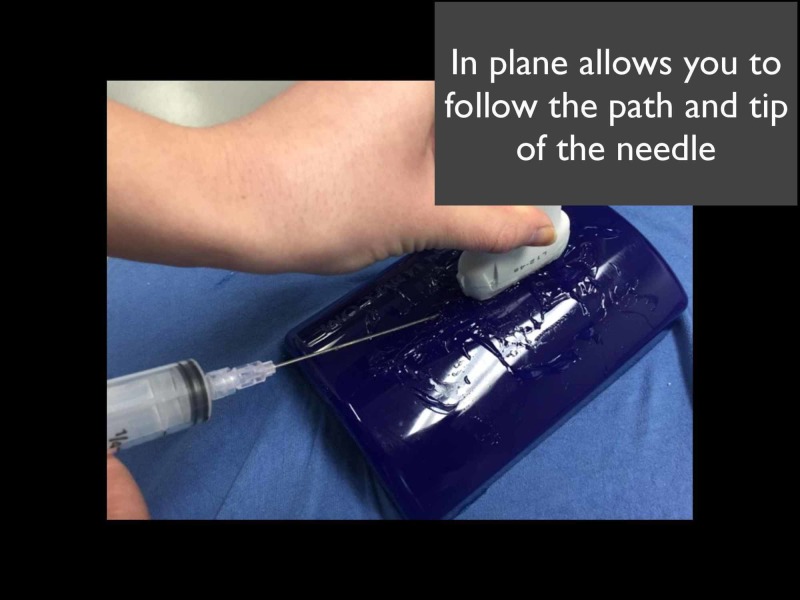
Image 3/12 of training presentation

**Figure 7:**
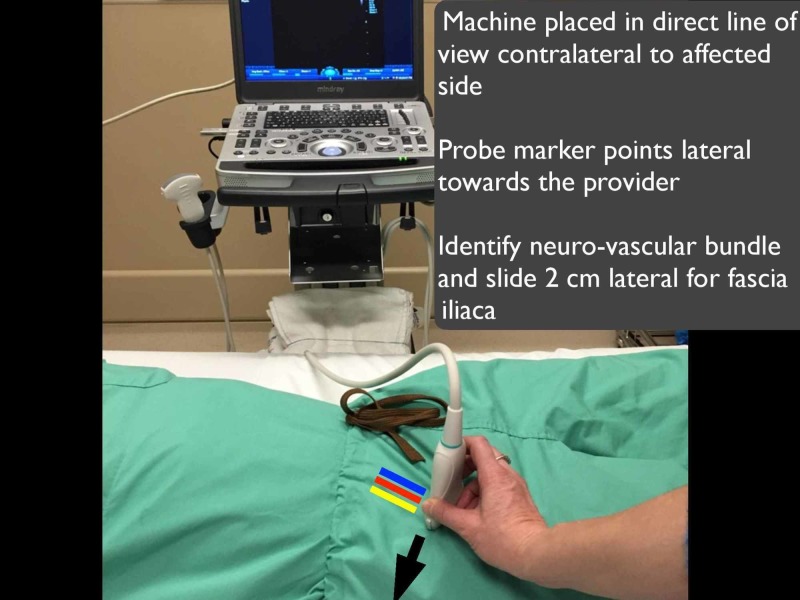
Image 4/12 of training presentation

**Figure 8:**
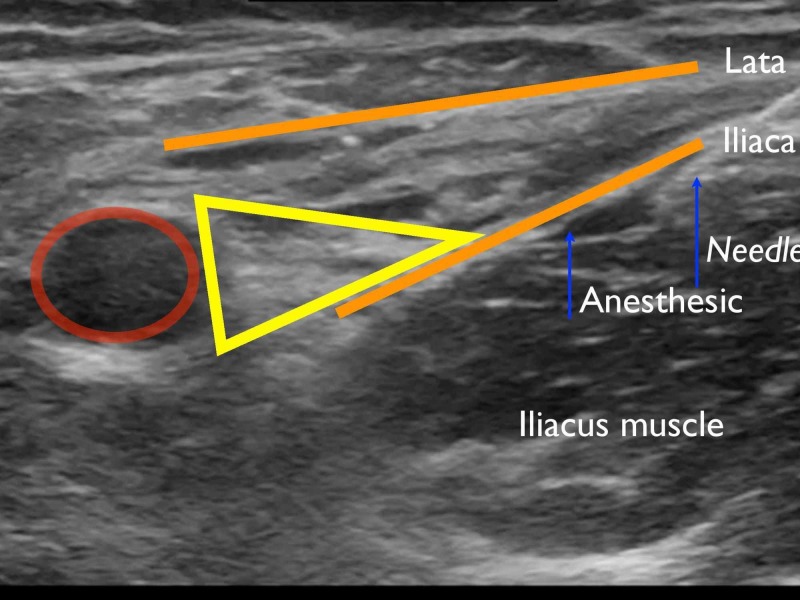
Image 5/12 of training presentation

**Figure 9:**
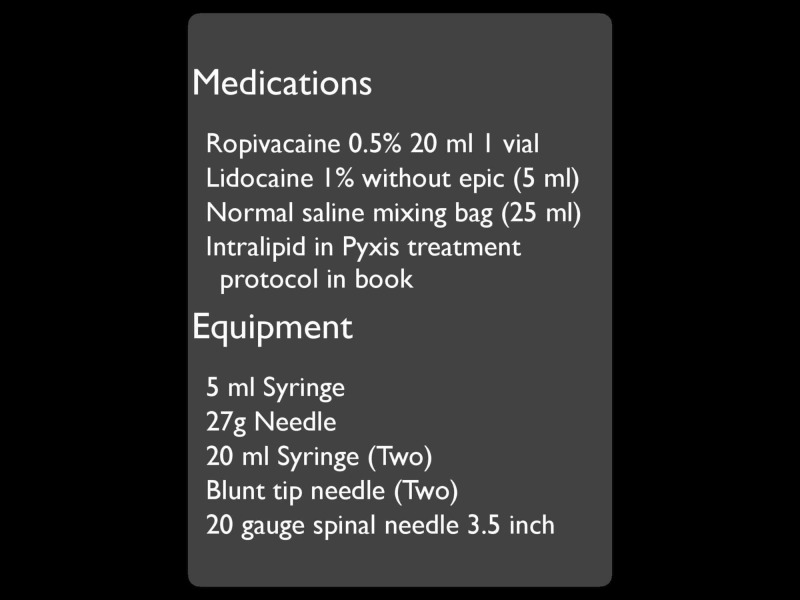
Image 6/12 of training presentation

**Figure 10:**
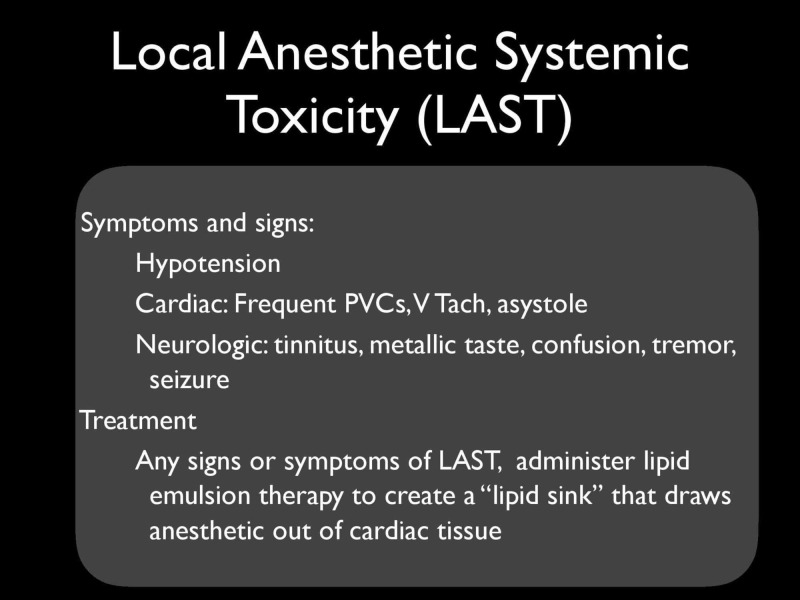
Image 7/12 of training presentation

**Figure 11:**
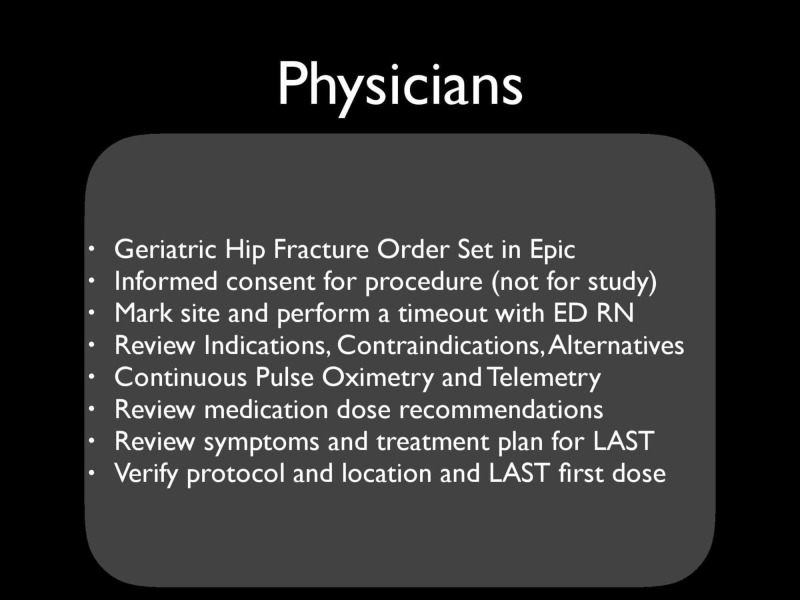
Image 8/12 of training presentation

**Figure 12:**
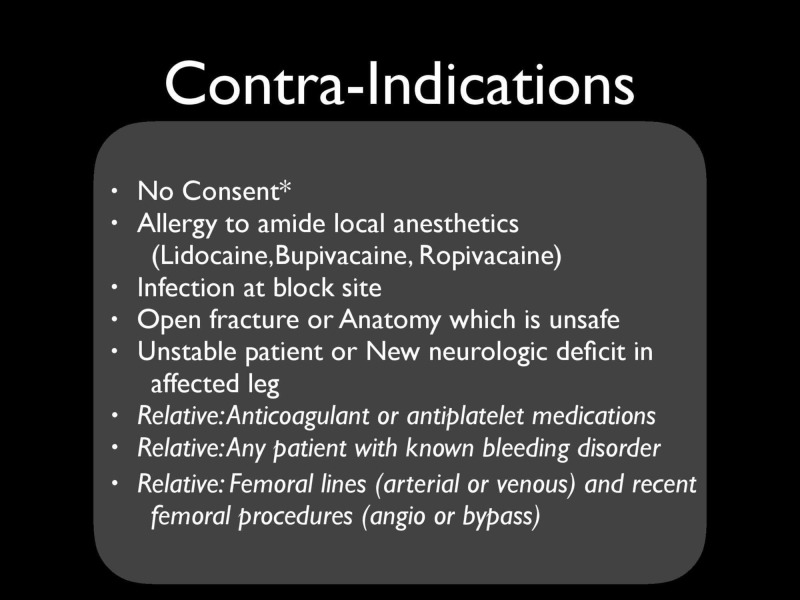
Image 9/12 of training presentation

**Figure 13:**
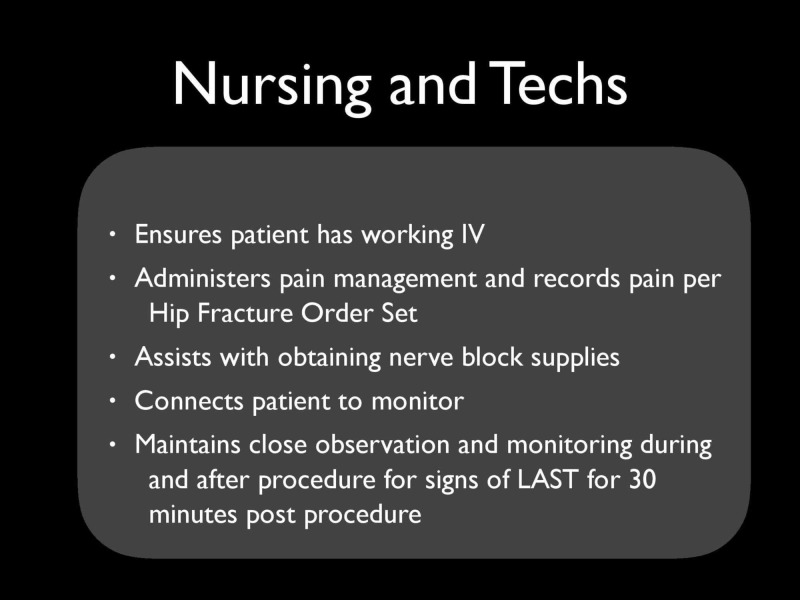
Image 10/12 of training presentation

**Figure 14:**
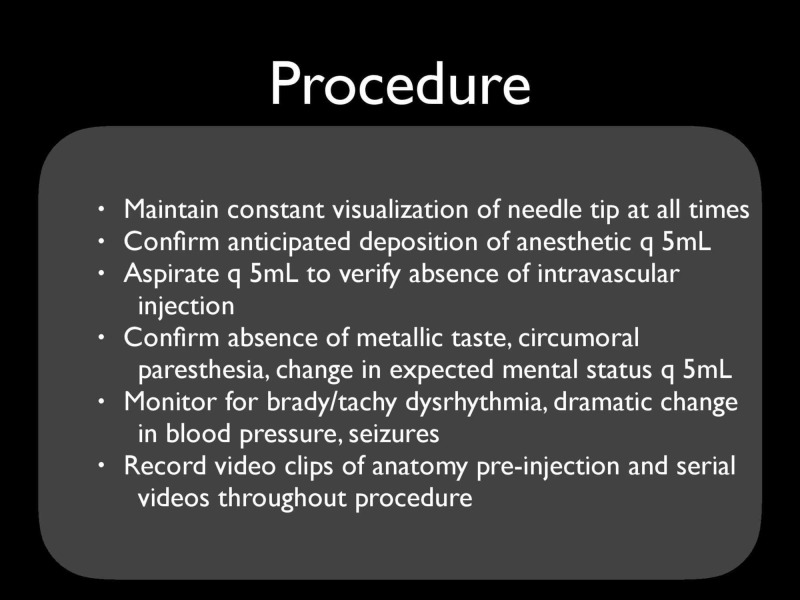
Image 11/12 of training presentation

**Figure 15:**
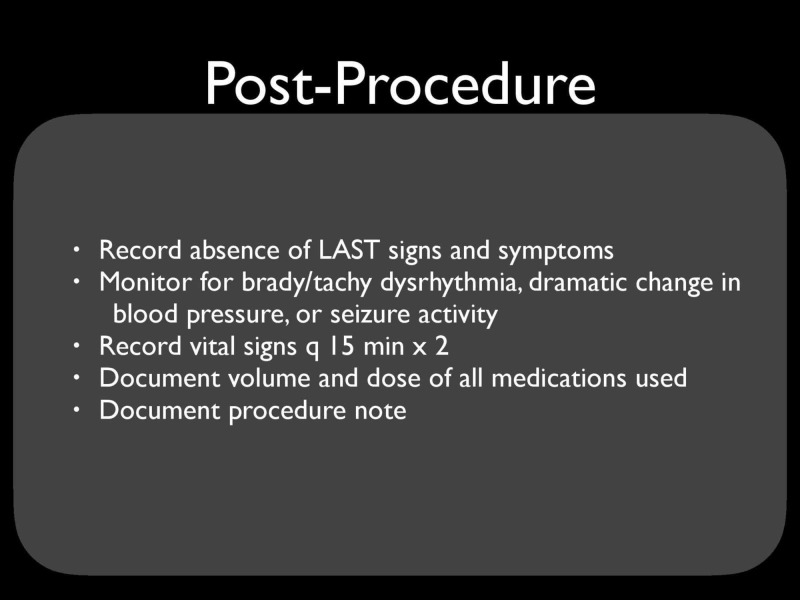
Image 12/12 of training presentation
